# Economic evaluation of finotonlimab plus bevacizumab as first-line therapy for advanced hepatocellular carcinoma

**DOI:** 10.1371/journal.pone.0349044

**Published:** 2026-05-14

**Authors:** XueYin Xu, Lian Tang, XiangHua Piao, ShaoQing Zhan, Yong Chen, PanFeng Feng

**Affiliations:** 1 Department of Pharmacy, Nantong First People’s Hospital, Southeast University, Nantong, Jiangsu, China; 2 Department of Pharmacy, Jiangwan Hospital of Shanghai Hongkou District, Shanghai, China; 3 Nantong Key Laboratory of Innovative Research on Rheumatology and Immunology‌‌, Nantong, Jiangsu, China; 4 Jiangsu Key Laboratory of New Drug Research and Clinical Pharmacy, Xuzhou Medical University, Xuzhou, Jiangsu, China; Hokkaido University: Hokkaido Daigaku, JAPAN

## Abstract

**Objective:**

To compare the cost-effectiveness of dual-agent group (finotonlimab combined with a bevacizumab biosimilar) (SCT510) versus sorafenib as first-line treatment for advanced hepatocellular carcinoma (HCC) from the perspective of the Chinese healthcare system.

**Methods:**

Based on the results of a Phase III clinical trial, a three-state partitioned survival model was constructed. The primary outcomes of the model included total costs, total quality-adjusted life years (QALYs), and the incremental cost-effectiveness ratio (ICER). Cost-effectiveness analysis was employed to evaluate the economic efficiency of the dual-agent group compared to the sorafenib group as first-line treatment for advanced HCC. The model cycle length was set at 3 weeks, with a time horizon of 10 years and a discount rate of 4.5%. The willingness-to-pay (WTP) threshold was set at three times China’s 2025 per capita gross domestic product (GDP) (299,400 CNY). One-way sensitivity analysis and probabilistic sensitivity analysis were conducted to assess the robustness of the results.

**Results:**

The ICER for the dual-agent group compared to the sorafenib group, calculated based on QALYs, was 859,053.76 CNY/QALY, which is higher than the WTP threshold (299,400 CNY). One-way sensitivity analysis indicated that parameters such as utility value in the PD state, utility value in the PFS state, the cost of finotonlimab and bevacizumab biosimilar had a significant impact on the ICER, while other parameters had minimal influence. The base-case analysis results were robust. Probabilistic sensitivity analysis showed that at a WTP threshold of 299,400 CNY, the probability of the dual-agent group being cost-effective was 0%. When the WTP threshold was approximately 842,000 CNY, the two groups had equal probability of being cost-effective. The probabilistic sensitivity analysis results were consistent with the base-case analysis.

**Conclusion:**

From the perspective of the Chinese healthcare system, finotonlimab combined with a bevacizumab biosimilar is not cost-effective as first-line treatment for advanced HCC.

## 1 Introduction

China is one of the countries with the heaviest burden of liver cancer worldwide. According to the latest statistical data, in 2022, the number of newly diagnosed liver cancer cases in China reached approximately 367,000, with more than 310,000 deaths. The incidence of liver cancer ranks fourth among all malignant tumors, while its mortality rate ranks second, especially in rural areas and among high-risk populations [1–5]. Due to the often subtle and easily overlooked early symptoms, the majority of patients are diagnosed at an advanced stage, significantly affecting treatment outcomes and survival. Currently, the overall five-year survival rate for liver cancer patients in China remains below 15%, indicating that the prevention and treatment landscape remains challenging [6–11].

Globally, sorafenib has long been regarded as the first-line standard of care for advanced hepatocellular carcinoma (HCC) [12]. However, the efficacy of this agent remains limited, with a median overall survival (mOS) generally shorter than one year [13]. Substantial evidence indicates that dual inhibition of the PD-L1/PD-1 axis and VEGF signaling pathway can synergistically enhance anti-tumor immune responses, offering a new strategic direction for the treatment of HCC [14,15]. Clinical studies have demonstrated that, in previously untreated patients with advanced hepatocellular carcinoma, toripalimab in combination with bevacizumab significantly prolongs progression-free survival and overall survival compared to sorafenib, along with a manageable safety profile [16]. Nevertheless, due to the high incidence and mortality of liver cancer, as well as factors such as heterogeneous regional regulatory approvals, restrictions in medical insurance coverage, and high treatment costs, there remains a substantial unmet clinical need [17,18]. Furthermore, owing to the high heterogeneity of hepatocellular carcinoma, not all patients benefit equally from current standard therapies. Those with severely impaired liver function, nonalcoholic fatty liver disease (NAFLD)-associated hepatocellular carcinoma, or contraindications to anti-angiogenic therapy are likely to have limited benefit [19,20]. Therefore, further optimization of treatment strategies and improvement of clinical outcomes remain important priorities in ongoing research.

Finotonlimab is a humanized IgG4 monoclonal antibody targeting PD-1, which has demonstrated anti-tumor activity in both preclinical studies and clinical trials [21].

A Phase III clinical trial conducted across 67 hospitals in China demonstrated that the combination of Finotonlimab and SCT510, a bevacizumab biosimilar, received authorization for use in China in June 2023, significantly extended median progression-free survival (7.1 months vs. 2.9 months) and median overall survival (22.1 months vs. 14.2 months) in patients, with a manageable safety profile [22]. Currently, there is a lack of pharmacoeconomic evaluation research on the Finotonlimab plus SCT510 treatment regimen in China. Therefore, from the perspective of the Chinese healthcare system, this study will apply pharmacoeconomic principles and methods to assess the cost-effectiveness of Finotonlimab in combination with SCT510 as a first-line treatment for advanced hepatocellular carcinoma, aiming to provide evidence-based support for healthcare policymakers, clinical teams, and patients when selecting treatment options for advanced hepatocellular carcinoma.

## 2 Method

### 2.1 Target population and treatment regimen

Patient data and treatment regimen information were derived from a clinical study [22], which was a multicenter, open-label, Phase III randomized controlled clinical trial conducted in China involving patients with advanced hepatocellular carcinoma. The Phase III study enrolled a total of 346 patients. Patient characteristics were derived from the phase III trial [22]. Briefly, enrolled patients were aged 18 years or older with histologically confirmed advanced hepatocellular carcinoma, Child-Pugh class A liver function, and Eastern Cooperative Oncology Group performance status 0–1.

In the Phase III study, patients were randomly assigned in a 2:1 ratio to either the dual-agent group (n = 230) or the sorafenib group (n = 116). Patients in the dual-agent group received intravenous infusions of finotonlimab (200 mg) plus the bevacizumab biosimilar SCT510 (15 mg/kg) every three weeks, whereas those in the sorafenib group received oral sorafenib (400 mg) twice daily. Treatment continued until any of the following occurred: disease progression, unacceptable toxicity, initiation of new antitumor therapy, death, or loss to follow-up. According to the phase III clinical trial [22] and the Chinese Guidelines for the Diagnosis and Treatment of Primary Liver Cancer (2024 Edition) [23], the subsequent treatment costs were based on the different drugs and proportions used in the clinical trial.

As this study is entirely based on previous research [22] and publicly available data, it does not include any new research involving human participants or animals by any of the authors, and therefore does not require approval from an independent ethics committee.

### 2.2 Model structure

**Fig 1:**
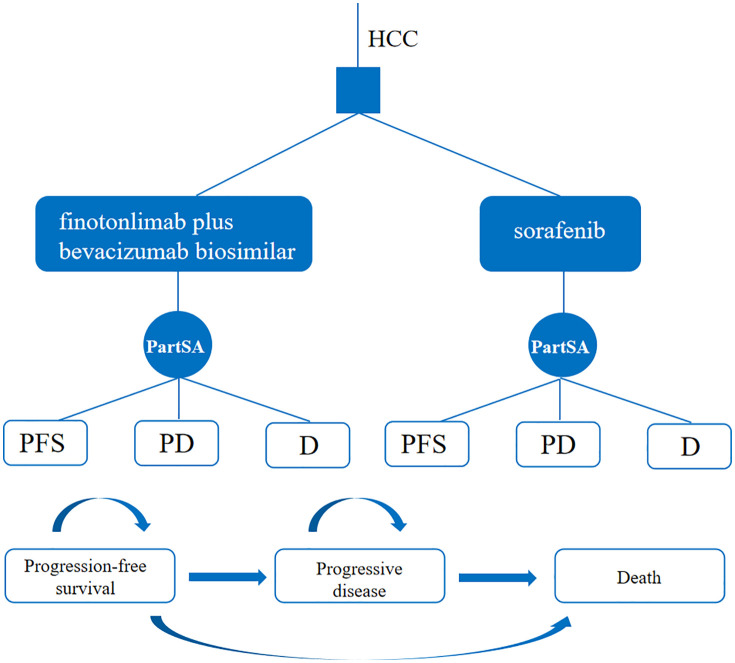
Partition survival model. PartSA, partitioned survival approach.

A three-state partitioned survival model was developed using TreeAge Pro 2022 software to simulate disease progression. The model comprised three health states: progression-free survival (PFS), progressive disease (PD), and death (D). All patients were assumed to enter the model in the PFS state. The cycle length was set to three weeks, aligned with the dosing regimen, and the time horizon was 10 years. The model structure is illustrated in Fig 1.

Key outcomes included total costs, incremental costs, quality-adjusted life years, incremental QALYs, and the incremental cost-effectiveness ratio (ICER). In accordance with the Chinese Guidelines for Pharmacoeconomic Evaluation 2025 [24], an annual discount rate of 4.5% was applied to both costs and health outcomes. The willingness-to-pay threshold was defined as three times China’s per capita gross domestic product. Based on the 2025 GDP figure, the WTP threshold was set at ¥299,400 per QALY.

### 2.3 Survival analysis

**Fig 2:**
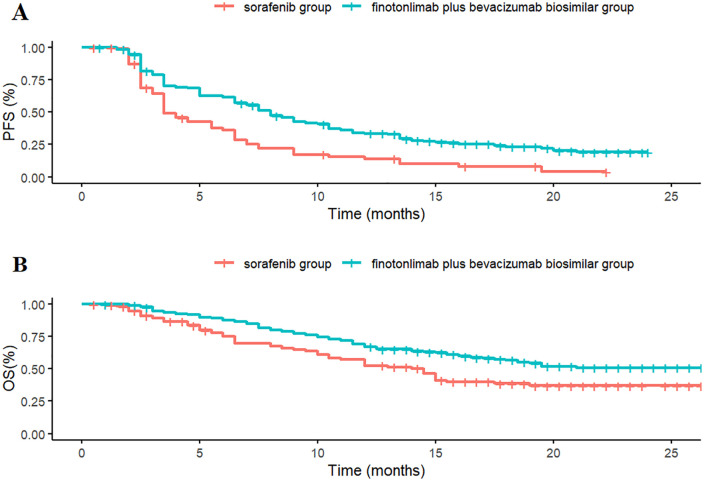
Optimal curve fitting extrapolation of two treatment schemes. **A.** Simulated PFS curve; **B.** Simulated OS curve.

Data points were extracted from the overall survival (OS) and progression-free survival (PFS) curves reported in the clinical trial using WebPlot Digitizer version 4.7. The extracted data were processed and reconstructed into individual patient data using R software version 4.4.1, and subsequently used to refit the OS and PFS curves (Fig 2). Several parametric distributions—including Exponential, Gompertz, Weibull, Log-logistic, and Lognormal—were fitted to the reconstructed patient-level time-to-event data. The best-fitting distribution was selected based on the lowest Akaike Information Criterion (AIC) and Bayesian Information Criterion (BIC) values [25], supplemented by visual inspection of the fitted curves. The results of the goodness-of-fit comparison are presented in Table 1. Ultimately, the Lognormal distribution was chosen to model both PFS and OS for each treatment group. The corresponding distribution parameters are summarized in Table 2.

**Table 1:** AIC and BIC of survival curve in two groups.

Survival curve	Model criteria	Exponetial	Gompertz	Weibull	Log-logistic	Lognormal
Dual-agent PFS curve	AIC	1032.664	1034.585	1027.754	1003.379	994.422
	BIC	1036.102	1041.462	1034.630	1010.255	1001.298
Sorafenib PFS curve	AIC	384.599	385.246	372.630	348.447	346.148
	BIC	387.352	390.753	378.137	353.955	351.655
Dual-agent OS curve	AIC	888.164	888.896	881.384	875.241	869.592
	BIC	891.602	895.772	888.260	882.117	876.468
Sorafenib OS curve	AIC	526.183	528.069	524.976	519.992	517.420
	BIC	528.936	533.576	530.483	525.499	522.927

**Table 2:** Parameter distribution of survival curve in two groups.

Survival curve	Optimal fitting distribution	Mean	SD
Dual-agent group PFS curve	Lognormal	2.11975	0.96625
Sorafenib group PFS curve	Lognormal	1.573341	0.703413
Dual-agent group OS curve	Lognormal	3.08907	1.09587
Sorafenib group OS curve	Lognormal	2.61494	1.09869

### 2.4 Costs and utilities

This study was conducted from the perspective of the Chinese healthcare system. Only direct medical costs were included in the cost estimation, encompassing drug costs, disease management costs [26], best supportive care expenses [27], end-of-life care costs [28], and costs associated with managing adverse events. Drug prices were based on the median 2025 tender prices across provinces in China, as published on Yaozh.com. Total treatment costs for both regimens were calculated according to the treatment duration observed in the clinical trial. For drugs dosed by body weight, a baseline weight of 59 kg was assumed [29]. Disease management costs included laboratory tests and imaging examinations. Data on adverse events (AEs) were obtained from the clinical study. Only AEs of Grade ≥3 severity with an incidence ≥5% in the original trial were incorporated. In clinical practice, Grade ≥3 AEs often lead to treatment discontinuation or switching; therefore, the model assumed a one-time cost for managing each such event, based on values derived from previously published literature [30–32]. Due to the lack of health utility data specific to the Chinese hepatocellular carcinoma population in the clinical trial, utility parameters were sourced from published studies: a utility value of 0.76 was applied for the progression-free survival state and 0.68 for the progressive disease state [33]. All model parameters and their distributions are summarized in Table 3.

**Table 3:** Model Parameters.

Variable	Baseline Value	Minimum	Maximum	Distribution	Reference
Cost/ CNY					
finotonlimab/mg	48.800	39.040	58.560	Gamma	Yaozhi
bevacizumab/mg	10.750	8.600	12.900	Gamma	Yaozhi
sorafenib/mg	0.075	0.060	0.090	Gamma	Yaozhi
Laboratory and imaging test	605.500	484.400	726.600	Gamma	[26]
Best supportive care	6653.380	5322.704	7984.056	Gamma	[27]
Terminal care	9313.370	7450.696	11176.044	Gamma	[28]
Proteinuria	1591.000	1272.800	1909.200	Gamma	[30]
Decreased platelet count	1505.920	1204.740	1807.100	Gamma	[31]
Hypertension	256.200	204.960	307.440	Gamma	[32]
Palmar-plantar erythrodysaesthesia syndrome	27.700	22.160	33.240	Gamma	[32]
Incidence rate of adverse reaction/ %					
Proteinuria (dual-agent group)	6.500	5.200	7.800	Beta	[22]
Decreased platelet count (dual-agent group)	7.000	5.600	8.400	Beta	[22]
Hypertension (dual-agent group)	8.700	6.960	10.440	Beta	[22]
Palmar-plantar erythrodysesthesia syndrome (sorafenib group)	6.900	5.520	8.280	Beta	[22]
Utilities					
PFS	0.760	0.608	0.912	Beta	[33]
PD	0.680	0.544	0.816	Beta	[33]
Others					
Weight	59.000	47.200	70.800	Normal	[29]
Discount rate/%	4.500	0.000	5.000	Beta	[24]

### 2.5 Scenario analysis

In this study, five parametric distributions were used to fit the PFS and OS curves, respectively, in order to evaluate their impact on the ICER. Additionally, the time horizon was set to 3, 4, 5, 6, 7, 8, 9, 10 and 15 years to further explore the influence of different simulation durations on the study results. Furthermore, changes in the ICER values were simulated under price reductions of 30%, 50% and 70% for finotonlimab and the bevacizumab biosimilar, respectively, to assess the impact of drug price negotiation on the study findings.

### 2.6 Sensitivity analysis

To verify the robustness of the base-case analysis results, both one-way sensitivity analysis and probabilistic sensitivity analysis were conducted. In the one-way sensitivity analysis, the value of a single variable was varied while keeping other parameters constant to assess its impact on the incremental cost-effectiveness ratio. The results were presented using a tornado diagram. If the upper and lower limits of a parameter were unknown, a variation of ±20% around the baseline value was assumed to define the plausible range. The outcomes of this analysis are illustrated in a tornado diagram. For the probabilistic sensitivity analysis, repeated sampling was performed based on the defined ranges and distribution types of the parameters, utilizing 1000 Monte Carlo simulations. Cost parameters were assigned a Gamma distribution, while the incidence of adverse events and utility parameters were modeled using Beta distributions. Cost-effectiveness acceptability curves and cost-effectiveness scatter plots were generated to evaluate the probability of each treatment strategy being cost-effective across a range of willingness-to-pay thresholds.

## 3 Results

### 3.1 The base case results

The base-case analysis showed that the total costs were ¥851,989.07 in the dual-agent group and ¥465,414.88 in the sorafenib group, with corresponding QALYs of 1.71 and 1.26, respectively. The dual-agent group provided an additional 0.45 QALYs at an increased cost of ¥386,574.19, resulting in an ICER of ¥859,053.76 per QALY. Since the ICER was above the pre-specified WTP threshold (Table 4), the dual-agent regimen was not considered cost-effective compared to sorafenib for the first-line treatment of advanced HCC.

**Table 4:** Baseline results.

Parameters	Dual-agent group	Sorafenib group
Total cost/CNY	851989.07	465414.88
Incremental cost/CNY	386574.19	–
Effect/QALYs	1.71	1.26
Incremental effect/QALYs	0.45	–
ICER, CNY/QALY	859053.76	–

### 3.2 Scenario analysis

Scenario analyses with varying time horizons demonstrated that as the simulation time extended, the ICER of the dual-agent group gradually decreased, with the magnitude of reduction diminishing over time. In all scenarios, the ICER remained above three times China’s 2025 per capita GDP (Table 5). Additional analyses using alternative parametric distributions to model PFS and OS also showed that all ICER values for the dual-agent group were above the WTP threshold (Table 6).

**Table 5:** Results of scenario analyses under different simulation time horizons.

Simulation time horizons	Group	Total cost/ CNY	Incremental cost/CNY	Effect/ QALYs	Incremental effect/QALYs	ICER, CNY/QALY
3 years	Dual-agent group	842181.84	381228.25	1.17	0.22	1732855.68
	Sorafenib group	460953.59		0.95		
4 years	Dual-agent group	846381.90	382071.26	1.34	0.27	1415078.74
	Sorafenib group	464310.64		1.07		
5 years	Dual-agent group	848637.97	383557.41	1.46	0.32	1198616.91
	Sorafenib group	465080.56		1.14		
6 years	Dual-agent group	849964.11	384665.40	1.54	0.36	1068515.00
	Sorafenib group	465298.71		1.18		
7 years	Dual-agent group	850796.42	385425.40	1.60	0.39	988270.26
	Sorafenib group	465371.02		1.21		
8 years	Dual-agent group	851345.52	385947.30	1.65	0.41	941334.88
	Sorafenib group	465398.22		1.24		
9 years	Dual-agent group	851722.27	386312.66	1.68	0.43	898401.53
	Sorafenib group	465409.61		1.25		
10 years	Dual-agent group	851989.07	386574.19	1.71	0.45	859053.76
	Sorafenib group	465414.88		1.26		
15 years	Dual-agent group	852585.08	387164.21	1.77	0.48	806592.10
	Sorafenib group	465420.87		1.29		

**Table 6:** Effects of different simulation distribution approaches on the ICER.

Distributions	Incremental cost/CNY	Incremental effect/QALYs	ICER, CNY/QALY
Exponential	383679.12	0.46	834085.04
Gompertz	383694.96	0.24	1598729.00
Weibull	382128.06	0.18	2122933.67
Log-logistic	365598.30	0.38	962100.79
Lognormal	386574.19	0.45	859053.76

Through price simulation, we predicted potential scenarios following the inclusion of finotonlimab and bevacizumab biosimilar into the National Reimbursement Drug List negotiations in China. We established a series of hypothetical drug price discount rates and recalculated the corresponding ICER values. The results showed that when drug prices were reduced by 30%, 50% and 70%, the corresponding ICER values were 774,887.78 CNY/QALY, 718,724.24 CNY/QALY, and 662,560.73 CNY/QALY, respectively (Table 7). Even under a 70% price reduction, the ICER remained significantly above the currently accepted willingness-to-pay threshold in China. This indicates that reducing drug prices alone is unlikely to make this regimen cost-effective under the current model assumptions.

**Table 7:** ICER values at different discount rates for drug prices.

Price discount rate	Group	Total cost/ CNY	Incremental cost/CNY	Effect/ QALYs	Incremental effect/QALYs	ICER, CNY/QALY
70%	Dual-agent group	814078.70	348699.50	1.71	0.45	774887.78
50%	Dual-agent group	788805.11	323425.91	1.71	0.45	718724.24
30%	Dual-agent group	763531.53	298152.33	1.71	0.45	662560.73
	Sorafenib group	465379.20		1.26		

### 3.3 One-way sensitivity analysis

**Fig 3:**
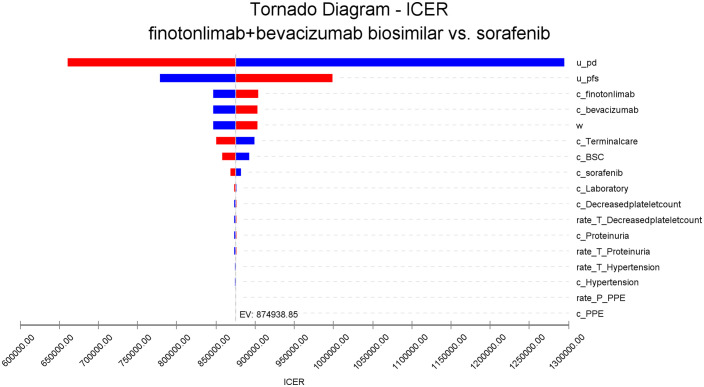
Tornado diagram for one-way sensitivity analysis.

The one-way sensitivity analysis indicated that parameters such as the utility value in the PD state, utility value in the PFS state, the cost of finotonlimab and bevacizumab biosimilar had the most substantial impact on the ICER. Other parameters exerted minimal influence. The results are summarized in the tornado diagram (Fig 3).

### 3.4 Probabilistic sensitivity analysis

**Fig 4:**
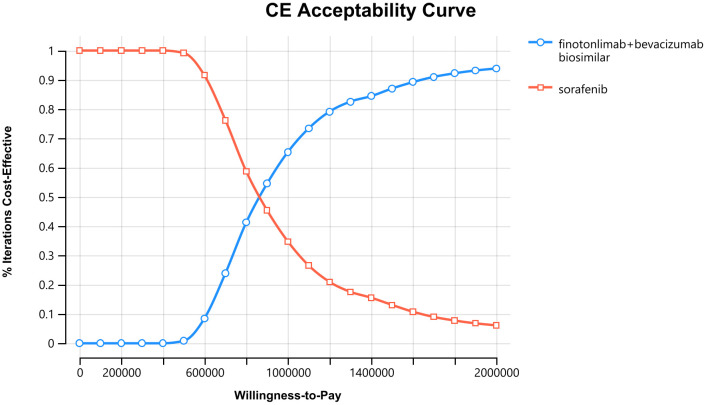
Acceptability curves.

**Fig 5:**
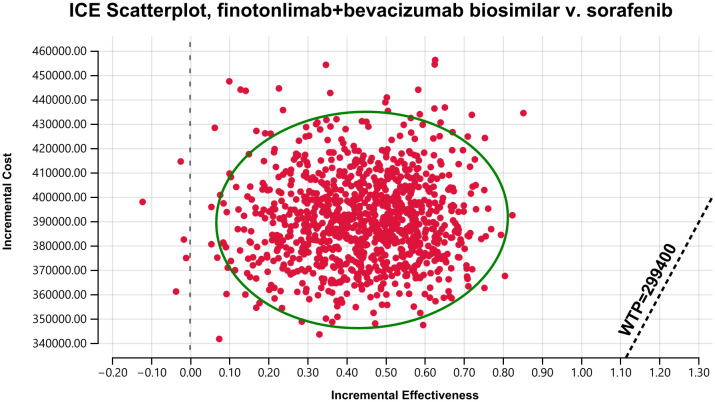
Cost-effective scatter plot. Results of Monte Carlo probabilistic sensitivity analysis showing incremental cost-effective of finotonlimab plus bevacizumab biosimilar versus sorafenib.

The probabilistic sensitivity analysis was presented using a cost-effectiveness acceptability curve (CEAC, Fig 4) and a cost-effectiveness scatter plot (Fig 5). The CEAC indicated that the probability of the dual-agent group being cost-effective increased with higher WTP thresholds, while the opposite trend was observed for sorafenib. At a WTP threshold of three times China’s 2025 per capita GDP (¥299,400), the probability of the dual-agent regimen being cost-effective was 0%. The two strategies had equal probability of being cost-effective at a WTP value of approximately ¥842,000. The scatter plot based on the second-order Monte Carlo simulation showed that all incremental cost-effectiveness points lay above the WTP threshold set at three times the per capita GDP, indicating that the dual-agent strategy had no economic advantage compared with sorafenib. These results were consistent with the base-case analysis.

## 4 Discussion‌‌

In China, the continuously rising costs of cancer treatment have led some patients to discontinue or even forgo therapy. Hence, selecting drugs with a cost-effectiveness advantage is particularly critical. By applying pharmacoeconomic evaluation methods to compare the economic efficiency of different treatment options, we can not only provide a reference for rational drug use in clinical practice but also help enhance the efficiency of healthcare insurance fund allocation [34]. Based on the NCT04560894 trial and from the perspective of the Chinese healthcare system, this study evaluated the cost-effectiveness of finotonlimab combined with a bevacizumab biosimilar (dual-agent group) versus sorafenib as first-line treatment for advanced hepatocellular carcinoma (HCC). The results showed an incremental cost-effectiveness ratio (ICER) of ¥859,053.76 per quality-adjusted life year (QALY), which was above the pre-defined willingness-to-pay (WTP) threshold. These findings suggest that the combination of finotonlimab and a bevacizumab biosimilar is not a cost-effective first-line treatment for advanced HCC.

One-way sensitivity analysis indicated that parameters such as the utility value in the PD state, utility value in the PFS state, the cost of finotonlimab and bevacizumab biosimilar had considerable impact on the results. Probabilistic sensitivity analysis demonstrated that at a WTP threshold of ¥299,400, the probability of the dual-agent regimen being cost-effective was 0%. When the WTP was approximately ¥842,000, both treatment strategies had equal probability of being cost-effective. The results of the probabilistic sensitivity analysis were consistent with the base-case analysis.

The results of this study indicated that although the combination of finotonlimab and a bevacizumab biosimilar extended overall survival compared with sorafenib, its incremental cost-effectiveness ratio was far above the current willingness-to-pay threshold in China, suggesting that this regimen was not cost-effective under current pricing. The findings of this study differed somewhat from existing economic evaluations supporting the cost-effectiveness of immuno-combination therapies for advanced hepatocellular carcinoma. For example, the atezolizumab plus bevacizumab regimen based on the IMbrave150 trial has become an international standard therapy, and subsequent economic evaluations conducted in China have generally shown that this regimen was cost-effective compared with sorafenib, with incremental cost-effectiveness ratios typically falling within acceptable thresholds [35]. Similarly, the sintilimab plus bevacizumab regimen based on the ORIENT-32 study was also demonstrated to be a cost-effective option in the Chinese context [36–38]. The higher ICER observed in this study was primarily attributable to the unclear pricing strategy and reimbursement status of finotonlimab as a novel PD-1 inhibitor, combined with uncertainties in the extrapolation of survival benefits, which prevented the regimen from demonstrating cost-effectiveness. This difference suggested that finotonlimab was not yet a preferred regimen in clinical practice, and further optimisation of pricing strategies or reduction of out-of-pocket costs through reimbursement negotiations was needed. This study provided preliminary evidence on the economic value of the finotonlimab combination regimen as first-line therapy for advanced hepatocellular carcinoma and served as a reference for future reimbursement decisions.

Consistent with other studies utilizing partitioned survival models, this study simulated the natural history of hepatocellular carcinoma and evaluated the cost-effectiveness of finotonlimab plus a bevacizumab biosimilar (dual-agent group) versus sorafenib as first-line therapy for advanced HCC within constrained healthcare resources. Nevertheless, several limitations should be acknowledged. First, patient data regarding progression-free survival and overall survival were derived from published clinical trials, which may introduce bias compared to real-world data. As this analysis is based on randomized controlled trials with strict inclusion/exclusion criteria and high patient adherence, the findings may not be fully generalizable to all patients encountered in routine clinical practice. Second, this study only considered direct medical costs, while direct non-medical costs and indirect costs were excluded. This may lead to an underestimation of the total actual treatment cost per patient. The costs associated with managing adverse events were sourced from published literature rather than real-world data, which might not adequately reflect the actual medical and economic context in China. Third, only severe adverse reactions (≥ Grade 3) with an incidence rate difference of ≥3% between the two treatment groups were included in the analysis. Not all AEs were considered, and the associated management costs may differ from actual clinical practice. However, one-way sensitivity analysis demonstrated that the cost of AE management had minimal impact on the overall results. Additionally, owing to the absence of disutility values for relevant adverse events, this study did not incorporate them into the analysis. Further investigation into their impact is warranted in future studies when reliable data become available. Fourth, compared with the Markov model, the partitioned survival model simplifies the disease course into three mutually exclusive health states, which makes it difficult to capture clinical realities such as subsequent treatments, disease recurrence, or complex transitions between states. This may affect the model’s ability to accurately reflect real-world clinical practice during the extrapolation period. Finally, in this study, five commonly used parametric distributions (Exponential, Gompertz, Weibull, Log-logistic, and Lognormal) were adopted for survival extrapolation, whereas seven-parameter distributions such as Gamma and Generalised Gamma were not included. Consequently, this may result in inadequate characterization of uncertainty in the tails of the survival curves.

Despite these limitations, this study thoroughly addressed uncertainties through extensive sensitivity analyses, which confirmed the robustness of the base-case findings. Therefore, the results still provide valuable references for clinical decision-making and health insurance reimbursement negotiations.

## 5 Conclusion

In conclusion, from the perspective of the Chinese healthcare system, and using a willingness-to-pay threshold of three times the Chinese GDP per capita, the finotonlimab plus bevacizumab biosimilar regimen is not a cost-effective first-line treatment option for patients with advanced hepatocellular carcinoma.

## References

[1] Bray F, Laversanne M, Sung H. Global cancer statistics 2022: GLOBOCAN estimates of incidence and mortality worldwide for 36 cancers in 185 countries. CA Cancer J Clin. 2024;74(3):229–63. 38572751 10.3322/caac.21834

[2] Han B, Zheng R, Zeng H. Cancer incidence and mortality in China, 2022. J Natl Cancer Cent, 2024;4(1):47–53. 39036382 10.1016/j.jncc.2024.01.006 PMC11256708

[3] Ye W, Wang J, Zheng J, Jiang M, Zhou Y, Wu Z. Association between Higher Expression of Vav1 in Hepatocellular Carcinoma and Unfavourable Clinicopathological Features and Prognosis. Protein Pept Lett. 2024;31(9):706–13. doi: 10.2174/0109298665330781240830042601 39301900

[4] Hajinourmohammadi A, Zargan J, Jafary H, Ebrahimi F. Evaluation of the Anti-Liver Cancer Activity of Protein Fractions Isolated from Adenium obesum Leaf Extract. Protein Pept Lett. 2025;32(10):742–55. doi: 10.2174/0109298665411024251015093155 41185505

[5] Mu R, Chang M, Feng C, Cui Y, Li T, Liu C, et al. Analysis of the Expression of PRDX6 in Patients with Hepatocellular Carcinoma and its Effect on the Phenotype of Hepatocellular Carcinoma Cells. Curr Genomics. 2024;25(1):2–11. doi: 10.2174/0113892029273682240111052317 38544826 PMC10964084

[6] González-Sánchez H, Castaño-García A, Celada-Sendino M, Flórez-Díez P, García-Calonge M, Rodríguez M, et al. Demographic and survival characteristics of untreated hepatocellular carcinoma patients: insights into the natural history and prognostic determinants. Rev Esp Enferm Dig. 2025;117(7):366–73. doi: 10.17235/reed.2025.11029/2024 39968627

[7] Chen W, Zheng R, Baade PD. Cancer statistics in China, 2015. CA Cancer J Clin. 2016;66(2):115–32. 10.3322/caac.21338 26808342

[8] Zeng H, Chen W, Zheng R, Zhang S, Ji JS, Zou X, et al. Changing cancer survival in China during 2003-15: a pooled analysis of 17 population-based cancer registries. Lancet Glob Health. 2018;6(5):e555–67. doi: 10.1016/S2214-109X(18)30127-X 29653628

[9] Xu W, Liao S, Hu Y. Upregulation of miR-3130-5p enhances hepatocellular carcinoma growth by suppressing ferredoxin 1: miR-3130-5p enhances HCC growth via inhibiting FDX1. Current Molecular Pharmacology. 2024;17:e18761429358008. doi: 10.2174/187414292358008 40103455

[10] Bansal S, Burman A, Sen A, Vachher M. Decoding Glycobiomarkers in Non-Alcoholic Steatohepatitis (NASH) and Related Hepatocellular Carcinoma (HCC). CP. 2024;22. doi: 10.2174/0115701646341608241025030547

[11] Martinez-Esquivias F, Martínez-Perez LA, Elena Iniguez-Munoz L, Mendez-Robles MD, Guzman-Flores JM. Bioinformatic Analysis and Molecular Docking to Elucidate the Anticancer Effect of Silver Nanoparticles in Hepatocellular Carcinoma. LDDD. 2024;21(18):4400–17. doi: 10.2174/0115701808352454241115065851

[12] Yang Y, Tang H, Mai C, Zhang X, Kuang J, Tang Y. Analysis of the Safety and Effectiveness of Lenvatinib + TACE-HAIC + PD-1 Inhibitor for Intermediate and Advanced Hepatocellular Carcinoma. LDDD. 2024;21(11):2035–45. doi: 10.2174/1570180820666230601113529

[13] Llovet JM, Ricci S, Mazzaferro V, Hilgard P, Gane E, Blanc J-F, et al. Sorafenib in advanced hepatocellular carcinoma. N Engl J Med. 2008;359(4):378–90. doi: 10.1056/NEJMoa0708857 18650514

[14] Finn RS, Bentley G, Britten CD, Amado R, Busuttil RW. Targeting vascular endothelial growth factor with the monoclonal antibody bevacizumab inhibits human hepatocellular carcinoma cells growing in an orthotopic mouse model. Liver Int. 2009;29(2):284–90. doi: 10.1111/j.1478-3231.2008.01762.x 18482274

[15] Finn RS, Qin S, Ikeda M, Galle PR, Ducreux M, Kim T-Y, et al. Atezolizumab plus Bevacizumab in Unresectable Hepatocellular Carcinoma. N Engl J Med. 2020;382(20):1894–905. doi: 10.1056/NEJMoa1915745 32402160

[16] Shi Y, Han G, Zhou J, Shi X, Jia W, Cheng Y, et al. Toripalimab plus bevacizumab versus sorafenib as first-line treatment for advanced hepatocellular carcinoma (HEPATORCH): a randomised, open-label, phase 3 trial. Lancet Gastroenterol Hepatol. 2025;10(7):658–70. doi: 10.1016/S2468-1253(25)00059-7 40409323

[17] Qian J, Jiang B, Qin Z. Knockdown of hsa_circ_0102231 impedes the progression of liver cancer through the miR-873–SOX4 axis. Current Gene Therapy. 2025;25(3):10. 38963113 10.2174/0115665232301878240627051455

[18] Sensi B, Angelico R, Toti L. Mechanism, potential, and concerns of immunotherapy for hepatocellular carcinoma and liver transplantation. Curr Mol Pharmacol. 2024;17:e18761429310703. 10.2174/0118761429310703240823045808 39225204

[19] The Chinese Chapter of the International Hepato-Pancreato-Biliary Association, Group of Liver Surgery, Surgical Society of Chinese Medical Association, Expert Committee on Liver Cancer, et al. Chinese multidisciplinary expert consensus on combined immunotherapy for hepatocellular carcinoma (2023 version). Chinese Journal of Hepatology. 2023;31(01):16–34. 36948846 10.3760/cma.j.cn501113-20221215-00602 PMC12814507

[20] Sangro B, Sarobe P, Hervás-Stubbs S, Melero I. Advances in immunotherapy for hepatocellular carcinoma. Nat Rev Gastroenterol Hepatol. 2021;18(8):525–43. doi: 10.1038/s41575-021-00438-0 33850328 PMC8042636

[21] Wang R, Zhang T, Lu Y, Lin Y, Kou S, Li X, et al. Antitumor activity of pegylated human interferon β as monotherapy or in combination with immune checkpoint inhibitors via tumor growth inhibition and dendritic cell activation. Cell Immunol. 2023;393–394:104782. doi: 10.1016/j.cellimm.2023.104782 37931572

[22] Zhao C, Zhang Y, Wang G, Zheng J, Chen W, Lu Z, et al. Finotonlimab (PD-1 inhibitor) plus bevacizumab (bevacizumab biosimilar) as first-tier therapy for late-stage hepatocellular carcinoma: a randomized phase 2/3 trial. Signal Transduct Target Ther. 2025;10(1):249. doi: 10.1038/s41392-025-02333-5 40769977 PMC12329032

[23] Department of Medical Administration NHC of the PRC. Guideline of diagnosis and treatment of primary liver cancer. Journal of Multidisciplinary Cancer. 2024;32(7):581–630.

[24] Wu J, Liu GE. China guidelines for pharmacoeconomic evaluation. Beijing: China market Press. 2025.

[25] You M, Chen R, Wu Q, Zhu W, He Y, Huang Y. Cost-effectiveness analysis of adebrelimab combined with chemotherapy for extensive-stage small cell lung cancer. Front Pharmacol. 2022;13:1019826. doi: 10.3389/fphar.2022.1019826 36386191 PMC9643856

[26] Chen QP, Shao MY, Cui HY. Cost-effectiveness analysis of durvalumab combined with tremelimumab in the first-line treatment of advanced hepatocellular carcinoma. Herald of Medicine. 2023;42(10):1492–7.

[27] Meng R, Zhou T, Shi FH. Cost-utility analysis of pembrolizuman in the second-line treatment of advanced hepatocellular carcinoma based on two models. China Pharmacv. 2021;32(22):2761–6.

[28] Liao W, Xu H, Hutton D. Cost-effectiveness analysis of durvalumab plus tremelimumab as first-line therapy in patients with unresectable hepatocellular carcinoma. Ther Adv Med Oncol. 2024;16:17588359241274625. 39301138 10.1177/17588359241274625 PMC11412210

[29] Commission PNHaS. Report on nutrition and chronic disease status of chinese residents. 2020. https://www.gov.cn/xinwen/2020-12/24/content_5572983.htm

[30] Hu Z, Guan X, Liu YJ. Optimization of cost-utility analysis with equal value of life-year gained: a case study of RAS-mutant metastatic colorectal cancer. China Pharmacy. 2024;35(18):2258–65.

[31] Xiang Z, Ma L, Fu Y, Pan Y. Cost-effectiveness analysis of first-line sintilimab plus chemotherapy vs. chemotherapy alone for unresectable advanced or metastatic gastric or gastroesophageal junction cancer in China. Front Pharmacol. 2024;15:1411571. doi: 10.3389/fphar.2024.1411571 39295936 PMC11408219

[32] Cai H, Zhang L, Li N, Zheng B, Liu M. Lenvatinib versus sorafenib for unresectable hepatocellular carcinoma: a cost-effectiveness analysis. J Comp Eff Res. 2020;9(8):553–62. doi: 10.2217/cer-2020-0041 32419473

[33] Leung HWC, Liu C-F, Chan ALF. Cost-effectiveness of sorafenib versus SBRT for unresectable advanced hepatocellular carcinoma. Radiat Oncol. 2016;11:69. doi: 10.1186/s13014-016-0644-4 27193904 PMC4870794

[34] Li X, Jia CF, Zheng Y. Pharmacoeconomic evaluation of trastuzumab deruxtecan versus chemotherapy in the second-line treatment of advanced breast cancer with HER-2 low expression. China Pharmacy. 2024;35(19):2383–90.

[35] Tseng C-Y, Tsai Y-W, Shiu M-N. Cost-effectiveness analysis of atezolizumab plus bevacizumab versus sorafenib in first line treatment for Chinese subpopulation with unresectable hepatocellular carcinoma. Front Oncol. 2023;13:1264417. doi: 10.3389/fonc.2023.1264417 38023232 PMC10663301

[36] Zhou T, Cao Y, Wang X, Yang L, Wang Z, Ma A, et al. Economic Evaluation of Sintilimab Plus Bevacizumab Versus Sorafenib as a First-line Treatment for Unresectable Hepatocellular Carcinoma. Adv Ther. 2022;39(5):2165–77. doi: 10.1007/s12325-022-02079-4 35296994

[37] Peng Y, Zeng X, Peng L, Liu Q, Yi L, Luo X, et al. Sintilimab Plus Bevacizumab Biosimilar Versus Sorafenib as First-Line Treatment for Unresectable Hepatocellular Carcinoma: A Cost-Effectiveness Analysis. Front Pharmacol. 2022;13:778505. doi: 10.3389/fphar.2022.778505 35222020 PMC8864224

[38] Li L, Yang S, Chen Y, Tian L, He Y, Wu B, et al. Immune Checkpoint Inhibitors Plus an Anti-VEGF Antibody as the First-Line Treatment for Unresectable Hepatocellular Carcinoma: A Network Meta-Analysis and Cost-Effectiveness Analysis. Front Pharmacol. 2022;13:891008. doi: 10.3389/fphar.2022.891008 35721168 PMC9198580

