# Supplementary material for: Economic evaluation of finotonlimab plus bevacizumab as first-line therapy for advanced hepatocellular carcinoma
Source: PLoS One. 2026 May 14;21(5):e0349044. doi: 10.1371/journal.pone.0349044 (PMC13175363; doi:10.1371/journal.pone.0349044)
Supplement: Attachment — Submitted filename: Response to comments.docx [file pone.0349044.s002.docx]

Reviewer #1: Thank you for your assigning me to review the submitted manuscript. This is not a methodologically rigorous economic evaluation, with significant limitations in data fitting, subsequent treatment costs, and disutility values. Therefore, the author should reconstruct the model and update the results.

Major comments:
1. Finotonlimab combined with a bevacizumab biosimilar yields superior efficacy at a higher cost, so why does the Log-logistic distribution yield a dominant result? This is an absolutely erroneous outcome. We seriously doubt the accuracy of the overall study.

Response: Thank you for your corrections. We have re-checked the model and recalculated the results. The revised results are shown in the table below. The ICER values under each distribution are all of a similar order of magnitude and all exceed the prespecified willingness-to-pay threshold, indicating that the conclusion is robust.

Table 6. Effects of different simulation distribution approaches on the ICER

| Distributions | Incremental cost/CNY | Incremental effect/QALYs | ICER, CNY/QALY |
| --- | --- | --- | --- |
| Exponential | 383679.12 | 0.46 | 834085.04 |
| Gompertz | 383694.96 | 0.24 | 1598729.00 |
| Weibull | 382128.06 | 0.18 | 2122933.67 |
| Log-logistic | 365598.30 | 0.38 | 962100.79 |
| Lognormal | 386574.19 | 0.45 | 859053.76 |

2. “Furthermore, owing to the high heterogeneity of hepatocellular carcinoma, not all patients benefit equally from current standard therapies.” Which groups of people? Please support your statement with relevant literature and data.

Response: We have followed your suggestion by adding the relevant citations and clearly indicating the specific population differences. Below is the revised paragraph along with the citations: “Furthermore, owing to the high heterogeneity of hepatocellular carcinoma, not all patients benefit equally from current standard therapies. Those with severely impaired liver function, nonalcoholic fatty liver disease (NAFLD)-associated hepatocellular carcinoma, or contraindications to anti-angiogenic therapy are likely to have limited benefit [19-20].”

19. The Chinese Chapter of the International Hepato-Pancreato-Biliary Association, Group of Liver Surgery, Surgical Society of Chinese Medical Association, Expert Committee on Liver Cancer, et al. Chinese multidisciplinary expert consensus on combined immunotherapy for hepatocellular carcinoma(2023 version). Chinese Journal of Hepatology. 2023, 31(01): 16-34.

20. Sangro B, Sarobe P, Hervás-Stubbs S, et al. Advances in immunotherapy for hepatocellular carcinoma. Nat Rev Gastroenterol Hepatol. 2021,18(8):525-543.

3. Please provide CHEERS 2020 in the supplementary materials.

Response: Thank you for your valuable suggestion. Following your advice, we have included the CHEERS 2022 checklist (Consolidated Health Economic Evaluation Reporting Standards 2022 statement) as supplementary material to present the details of this economic evaluation more completely and in a more standardized manner.
4. Removing the inclusion and exclusion criteria of clinical trials, as these are unrelated to the study methodology. A brief description of patient characteristics is sufficient.

Response: Thank you for your valuable suggestion. Following your advice, we have removed the detailed inclusion and exclusion criteria of the clinical trial that were not directly relevant to the research methods from the original manuscript, retaining only a brief description of the patient characteristics, as follows: "Patient characteristics were derived from the phase III trial [22]. Briefly, enrolled patients were aged 18 years or older with histologically confirmed advanced hepatocellular carcinoma, Child-Pugh class A liver function, and Eastern Cooperative Oncology Group performance status 0–1."
5. To our knowledge, the subsequent treatment regimen recommended in the CSCO 2024 guidelines does not best supportive care. Please calculate the subsequent treatment costs based on the different drugs (Immunotherapy, Targeted therapy, Chemotherapy) and proportions used in clinical trials (Supplementary Table 4).

Response: In accordance with your suggestion, we have re-estimated the subsequent treatment costs as follows:

1. Based on the distribution of subsequent treatments reported in the clinical trial (Supplementary Table 4), we focused on three drug therapy regimens that are widely used in clinical practice, have clearly defined costs, and exert a significant impact on the results: Immunotherapy, Targeted therapy, and Chemotherapy. The costs of these regimens have been accurately incorporated into the calculation of cycle costs for the progressed disease state, where the drug treatment cost is derived as the proportion of patients receiving subsequent therapy multiplied by the drug price multiplied by the number of cycles.
2. In the analysis, we observed that a small proportion of patients received Local therapy, Radiotherapy, or Others. After careful evaluation, we decided to temporarily exclude these regimens from the present analysis, primarily based on the following two considerations: a) Low patient selection proportion: The applicable patient population for these treatment regimens is relatively limited, accounting for a low proportion of the overall cohort; b) Difficulty in uniform quantification of costs: The costs of these regimens vary considerably across different hospitals and individual patients. The lack of a reliable and representative standard cost for estimation means that including them could introduce greater uncertainty.
3. Updated analysis results: We re-conducted the cost-effectiveness analysis using the revised model that incorporates the costs of the above-mentioned post-progression systemic drug therapies. The revised results indicate that, under the willingness-to-pay (WTP) threshold established in this study, finotonlimab plus bevacizumab biosimilar is not cost-effective compared with sorafenib for the treatment of advanced HCC.
   6. Why choose the partitioned survival model rather than the Markov model? What are the differences between them?

Response: Thank you for your valuable comment. The partitioned survival model and the Markov model are both commonly used decision-analytic modeling approaches in pharmacoeconomic evaluations. Differences exist between the two: a partitioned survival model typically divides the disease course into three states—progression-free, progressed, and death—and directly uses Kaplan-Meier (KM) curves for progression-free survival (PFS) and overall survival (OS) obtained from clinical trials to calculate the proportion of patients in each state at each time point. The model does not rely on transition probabilities between states but derives outcomes directly through survival functions. In contrast, a Markov model requires explicit specification of transition probabilities between health states, which often need to be estimated from survival data through parametric model fitting, and predicts outcomes by simulating the distribution of a cohort across states over time. The Markov model has advantages when there are multiple health states or when transitions between states are complex.

In this study, we chose the partitioned survival model primarily for the following reasons: (1) Key differences in cost-effectiveness analyses often arise from costs incurred during treatment. The partitioned survival model clearly distinguishes between progression-free and progressed states, directly reflecting the temporal distribution of treatment costs. (2) The partitioned survival model makes full use of the original KM data; we only need to perform parametric extrapolation of the tail portions of the OS and PFS curves, without additional assumptions regarding transition probabilities between states, thereby reducing the risk introduced by the specification of transition probabilities. (3) The partitioned survival model is suitable when clinical trials directly provide KM curves for PFS and OS and when the number of disease states is limited. It has become one of the mainstream methods in contemporary pharmacoeconomic evaluations of oncological therapies. Many previous pharmacoeconomic studies have also employed this model [1–3], and the model specification in our study is consistent with the aforementioned literature, providing a sound methodological basis.

Of course, any model choice has its limitations. We have also addressed this in the Discussion section, as follows: “Compared with the Markov model, the partitioned survival model simplifies the disease course into three mutually exclusive health states，which makes it difficult to capture clinical realities such as subsequent treatments, disease recurrence, or complex transitions between states. This may affect the model’s ability to accurately reflect real-world clinical practice during the extrapolation period.”。

[1] Tseng CY, Tsai YW, Shiu MN. Cost-effectiveness analysis of atezolizumab plus bevacizumab versus sorafenib in first line treatment for Chinese subpopulation with unresectable hepatocellular carcinoma. Front Oncol. 2023;13:1264417.

[2] Zhou T, Cao Y, Wang X, et al. Economic Evaluation of Sintilimab Plus Bevacizumab Versus Sorafenib as a First-line Treatment for Unresectable Hepatocellular Carcinoma. Adv Ther. 2022 ;39(5):2165-2177.

[3] Li L, Yang S, Chen Y, et al. Immune Checkpoint Inhibitors Plus an Anti-VEGF Antibody as the First-Line Treatment for Unresectable Hepatocellular Carcinoma: A Network Meta-Analysis and Cost-Effectiveness Analysis. Front Pharmacol. 2022;13:891008.

7. According to the Economic Evaluation Guidelines 2025, the discount rate is 4.5%, with a range of 0-5%.

Response: Thank you for your valuable comment. Regarding the issue that the discount rate should follow the Economic Evaluation Guidelines 2025, we have carefully verified and made corresponding revisions. The basis for the discount rate setting has been updated in the Methods section of the main text, and the discount rate in the model has been adjusted to 4.5%. The updated results show that, under the willingness-to-pay (WTP) threshold established in this study, finotonlimab plus bevacizumab biosimilar is not cost-effective compared with sorafenib for the treatment of advanced hepatocellular carcinoma (HCC).
8. Please update the GDP per capita to 2025 (￥99800).

Response: Thank you for your careful correction. The per capita GDP data cited in the original manuscript were from 2024. Based on the latest 2025 data (¥99,800), we have updated all relevant per capita GDP values in the main text to ¥99,800 and recalculated the threshold for the incremental cost-effectiveness ratio (ICER). Under the updated threshold, the finotonlimab plus bevacizumab regimen is not cost-effective.

9. Typically, seven-parameter survival models are employed for data fitting. Please add Gamma and Generalised Gamma, and present the AIC and BIC values for different parameter survival models in the supplementary materials.

Response: Thank you for your suggestion to include the Gamma and Generalised Gamma distributions. After careful consideration, we have decided to retain the five commonly used parametric distributions (Exponential, Gompertz, Weibull, Log-logistic, and Lognormal) for survival extrapolation in this study, primarily for the following reasons:

(1) Most pharmacoeconomic evaluations of advanced hepatocellular carcinoma employ these five parametric distributions for survival extrapolation, whereas the use of Gamma and Generalised Gamma distributions is relatively rare [1]. Furthermore, in actual NICE single technology appraisals of oncological drugs, Exponential, Weibull, Log-logistic, Lognormal, and Gompertz are the most commonly used parametric distributions, while Gamma and Generalised Gamma are not routine choices due to their larger number of parameters and questionable extrapolation stability [2]. A study published in ASCO also showed that in survival extrapolation practices for oncological pharmacoeconomic evaluations, Weibull (33%), Log-logistic (32%), and Log-normal (27%) are the three most widely applied distributions, whereas the frequency of use of Gamma and Generalised Gamma is significantly lower [3]. (2) Some economic modeling software (e.g., earlier versions of TreeAge) offers limited support for the Generalised Gamma distribution, particularly when the Q parameter is negative, which may lead to computational errors. To ensure model reproducibility and a smooth analytical process, we adopted the five-parameter distributions.

Following your suggestion, we have added the following content to the Limitations section: "In this study, five commonly used parametric distributions (Exponential, Gompertz, Weibull, Log-logistic, and Lognormal) were adopted for survival extrapolation, whereas seven-parameter distributions such as Gamma and Generalised Gamma were not included. Consequently, this may result in inadequate characterization of uncertainty in the tails of the survival curves."

[1] Zou H, Xue Y, Chen X,et al. Comparative analysis of disease modelling for health economic evaluations of systemic therapies in advanced hepatocellular carcinoma. PLoS One. 2023,18(10):e0292239.
[2] Connock M, Hyde C, Moore D. Cautions regarding the fitting and interpretation of survival curves: examples from NICE single technology appraisals of drugs for cancer. Pharmacoeconomics. 2011,29(10):827-37.
[3] Roze S, Bertrand N, Eberst L, et al. Projecting overall survival in health-economic models: uncertainty and maturity of data. Curr Med Res Opin. 2023,39(3):367-374.

10. What does “Laboratory and imaging tests” include? Please specify the specific tests and costs (Blood tests, CT scans, bone scans, etc.).

Response: Thank you for your thorough review. It is true that the original reference we cited in the manuscript does not list the specific items or unit prices for "laboratory and imaging examinations." According to the Guidelines for the Diagnosis and Treatment of Primary Liver Cancer (2024 Edition), such examinations typically include laboratory tests such as blood routine, liver and kidney function, and tumor markers, as well as imaging examinations such as contrast-enhanced CT or MRI. To ensure the robustness of the study, we have incorporated these examination costs into the sensitivity analysis by defining a reasonable variation range (e.g., ±20%) to assess the impact of their uncertainty on the final results.
11. How are the costs of adverse events incorporated into the model?

Response: In the cost-effectiveness analysis model of this study, adverse event (AE) costs were included as a component of direct medical costs. Based on the incidence rates of AEs observed in the two treatment arms of the clinical trial, we focused on grade ≥3 serious AEs with an incidence rate ≥5%. The cost was calculated as follows: Total AE cost = Σ (incidence rate of each AE × unit management cost for that AE). In the partitioned survival model, AE costs were incorporated as one-time event costs in the model cycle in which the AE occurred. To account for the uncertainty in AE incidence rates and management costs, we varied these parameters within ±20% of their base-case values and performed 1,000 Monte Carlo simulations. The analysis results showed that, within a reasonable range of variation in AE costs, the main conclusions of this study remained robust.

12. Why are the utility values derived from Reference 22 rather than published real-world studies? Why were disutility values associated with adverse events not considered? A rigorous economic evaluation should incorporate disutility values.

Response: Thank you for your valuable suggestion.

(1) Since the phase III clinical trial on which this study is based did not disclose patient health utility values, and currently there are relatively limited large-scale, prospective real-world studies on health utility values specifically for Chinese patients with advanced hepatocellular carcinoma, with existing studies varying in population characteristics, measurement instruments, and scoring algorithms, direct inclusion of such values might introduce heterogeneity bias. Therefore, we adopted a set of PFS and PD state utility values that have been highly cited in published literature [1–3]. To ensure the robustness of the study conclusions, we tested the utility values within a range of ±20% of their base-case values in the probabilistic sensitivity analysis. The results showed that even with wide variations in the utility values, the fluctuation in the ICER was limited, and the study conclusions remained consistently stable.

[1] Luo J, Li Z, Ge J, et al. Nivolumab plus ipilimumab versus lenvatinib or sorafenib as first-line treatment for unresectable hepatocellular carcinoma: A cost-effectiveness analysis [J]. Cancer. 2026,132(3):e70259.
[2] Wu Q, Qin Y, Li Q. Nivolumab plus ipilimumab versus lenvatinib or sorafenib for US and Chinese patients with unresectable hepatocellular carcinoma: a cost-effectiveness analysis [J]. Front Public Health. 2026,13:1726477.
[3] Zhao M, Pan X, Yin Y, et al. Cost-Effectiveness Analysis of Five Systemic Treatments for Unresectable Hepatocellular Carcinoma in China: An Economic Evaluation Based on Network Meta-Analysis. Front Public Health. 2022,10:869960.

(2) We fully agree that a rigorous pharmacoeconomic evaluation should adequately account for the impact of adverse events on health-related quality of life and incorporate corresponding disutility values. We conducted a literature search for the four adverse events of concern in this study. For proteinuria and palmar-plantar erythrodysesthesia syndrome, no directly available disutility values were identified in the published literature. Similarly, for thrombocytopenia and hypertension, disutility values that perfectly matched the intervention regimen of this study were lacking. Therefore, directly incorporating these values might introduce uncertainty and bias. In the Limitations section of the Discussion, we have added the following statement: "Owing to the absence of disutility values for relevant adverse events, this study did not incorporate them into the analysis. Further investigation into their impact is warranted in future studies when reliable data become available."
13. What is the difference between 1000 and 10,000 times second-order Monte Carlo simulations? Why choose 1000 times?

Response: Thank you for your question. Second-order Monte Carlo simulation is typically used in probabilistic sensitivity analysis, where the outer loop simulates the uncertainty in parameter distributions and the inner loop simulates random error at the individual level. The main difference between 1,000 and 10,000 iterations is that in most pharmacoeconomic evaluations, 1,000 outer-loop iterations are generally sufficient to achieve stability in the incremental cost-effectiveness ratio (ICER) with good reproducibility. While increasing the number of iterations to 10,000 can slightly improve the stability of estimation precision, it substantially increases computation time with limited marginal benefit.

This study chose 1,000 second-order Monte Carlo simulations for the following reasons: (1) According to guideline recommendations, 1,000 simulations in probabilistic sensitivity analysis are generally considered an acceptable minimum number of iterations, allowing for robust statistical inference while controlling computational costs. (2) One thousand simulations are widely used in published pharmacoeconomic evaluations of oncological therapies. For example, a study published in Cancer (2026) titled "Nivolumab plus ipilimumab versus lenvatinib or sorafenib as first-line treatment for unresectable hepatocellular carcinoma: A cost-effectiveness analysis" explicitly stated that "1,000 Monte Carlo simulations were performed." Similarly, a study published in Frontiers in Public Health (2026) titled "Nivolumab plus ipilimumab versus lenvatinib or sorafenib for US and Chinese patients with unresectable hepatocellular carcinoma: a cost-effectiveness analysis" also indicated that "For the probabilistic sensitivity analysis (PSA), 1,000 Monte Carlo simulations were performed."
14. Please reconstruct the model, as the current results are absolutely incorrect.

Response: Thank you for your rigorous review. We have conducted a comprehensive and systematic re-examination and reconstruction of the model. The results of the revised model analysis show that the incremental cost-effectiveness ratios (ICERs) under all parametric distributions are positive, indicating that the conclusions are robust and reliable. The corresponding results and methodological descriptions have been updated in the revised manuscript.
15. The current discussion does not meet the standards outlined in the economic evaluation guidelines(CHEERS 2020). The author should explain why the novel treatment regimen is or is not cost-effective. The author should briefly review existing economic evaluations related to advanced hepatocellular carcinoma, identifying the optimal/preffered treatment regimen in available economic evaluations and comparing it with the findings of this study.

Response: Thank you for your valuable comments. We have comprehensively revised the Discussion section in accordance with the CHEERS 2020 checklist. The main additions are described as follows:

(1) We have explicitly stated in the Discussion that although finotonlimab plus bevacizumab biosimilar provides a gain in overall survival (0.45 QALYs) compared with sorafenib, its incremental cost is as high as approximately ¥386,574.19, resulting in an ICER far above the prespecified willingness-to-pay threshold. Therefore, this combination regimen does not have a cost-effectiveness advantage at the current price level. We further analyzed the key factors influencing the ICER, including drug prices, the choice of PFS/OS extrapolation curves, and the sensitivity of utility parameters. Sensitivity analyses showed that even under different parameter assumptions, the ICER generally remained above the threshold, indicating that the conclusion is robust.
(2) A review and comparison of existing economic evaluations of advanced hepatocellular carcinoma have been added to the Discussion section, with the following new content inserted: “The results of this study indicated that although the combination of finotonlimab and a bevacizumab biosimilar extended overall survival compared with sorafenib, its incremental cost-effectiveness ratio was far above the current willingness-to-pay threshold in China, suggesting that this regimen was not cost-effective under current pricing. The findings of this study differed somewhat from existing economic evaluations supporting the cost-effectiveness of immuno‑combination therapies for advanced hepatocellular carcinoma. For example, the atezolizumab plus bevacizumab regimen based on the IMbrave150 trial has become an international standard therapy, and subsequent economic evaluations conducted in China have generally shown that this regimen was cost-effective compared with sorafenib, with incremental cost-effectiveness ratios typically falling within acceptable thresholds [35]. Similarly, the sintilimab plus bevacizumab regimen based on the ORIENT‑32 study was also demonstrated to be a cost‑effective option in the Chinese context [36-38]. The higher ICER observed in this study was primarily attributable to the unclear pricing strategy and reimbursement status of finotonlimab as a novel PD‑1 inhibitor, combined with uncertainties in the extrapolation of survival benefits, which prevented the regimen from demonstrating cost‑effectiveness. This difference suggested that finotonlimab was not yet a preferred regimen in clinical practice, and further optimisation of pricing strategies or reduction of out‑of‑pocket costs through reimbursement negotiations was needed. This study provided preliminary evidence on the economic value of the finotonlimab combination regimen as first‑line therapy for advanced hepatocellular carcinoma and served as a reference for future reimbursement decisions.”

[35] Tseng CY, Tsai YW, Shiu MN. Cost-effectiveness analysis of atezolizumab plus bevacizumab versus sorafenib in first line treatment for Chinese subpopulation with unresectable hepatocellular carcinoma. Front Oncol. 2023;13:1264417.

[36] Zhou T, Cao Y, Wang X, et al. Economic Evaluation of Sintilimab Plus Bevacizumab Versus Sorafenib as a First-line Treatment for Unresectable Hepatocellular Carcinoma. Adv Ther. 2022 ;39(5):2165-2177.

[37] Peng Y, Zeng X, Peng L, et al. Sintilimab Plus Bevacizumab Biosimilar Versus Sorafenib as First-Line Treatment for Unresectable Hepatocellular Carcinoma: A Cost-Effectiveness Analysis. Front Pharmacol. 2022;13:778505.

[38] Li L, Yang S, Chen Y, et al. Immune Checkpoint Inhibitors Plus an Anti-VEGF Antibody as the First-Line Treatment for Unresectable Hepatocellular Carcinoma: A Network Meta-Analysis and Cost-Effectiveness Analysis. Front Pharmacol. 2022;13:891008.

Reviewer #2: This manuscript presents a well-conducted pharmacoeconomic analysis suggesting finotonlimab plus bevacizumab biosimilar is cost-effective versus sorafenib for advanced HCC in China. While the methodological framework is generally appropriate, several critical issues must be addressed to ensure robustness and transparency. Below are the major and minor concerns:
1、Drug Cost Assumptions Need Validation：The model assumes full drug costs without accounting for possible charity drug programs during the trial (2022–2025), as both Finotonlimab and bevacizumab biosimilar were unapproved in China at the time. Maybe sensitivity analysis are needed, try to explore reduced pricing scenarios if possible (e.g., discount rates negotiated post-approval).

Response: Thank you for pointing out the potential limitations in the drug cost assumptions. To examine the impact of drug price assumptions on the study conclusions, we conducted the following scenario analyses. Referring to the average price reductionof PD-1 inhibitors in recent Chinese national negotiations, we set three price reduction scenarios: drug prices were reduced by 30%, 50%, and 70%. Under each of these price reduction scenarios, the incremental cost-effectiveness ratio (ICER) was recalculated. The results showed that under the 30% price reduction scenario, the ICER decreased to approximately ¥774,887.78/QALY, which remained significantly above the threshold. Under the 50% price reduction scenario, the ICER decreased to approximately ¥718,724.24/QALY, still above the threshold. Under the 70% price reduction scenario, the ICER decreased to approximately ¥662,560.73/QALY, still above the threshold. Even with a 70% price reduction, the ICER remained substantially higher than the currently accepted willingness-to-pay threshold in China. This indicates that reducing drug prices alone is unlikely to confer a cost-effectiveness advantage to this regimen under the current model assumptions. We have added relevant content in the Discussion section, emphasizing the impact of drug pricing strategies on cost-effectiveness, and suggest that future studies should further update the evaluation once drug prices are clarified.

Table 7. ICER values at different discount rates for drug prices

| Price discount rate | Group | Total cost/  CNY | Incremental cost/CNY | Effect/  QALYs | Incremental effect/QALYs | ICER, CNY/QALY |
| --- | --- | --- | --- | --- | --- | --- |
| 70% | Dual-agent group | 814078.70 | 348699.50 | 1.71 | 0.45 | 774887.78 |
| 50% | Dual-agent group | 788805.11 | 323425.91 | 1.71 | 0.45 | 718724.24 |
| 30% | Dual-agent group | 763531.53 | 298152.33 | 1.71 | 0.45 | 662560.73 |
|  | Sorafenib group | 465379.20 |  | 1.26 |  |  |

2、Incomplete Adverse Event (AE) Cost Capture：Serious AEs (≥Grade 3) reported in the Phase III trial (e.g., 27 cases of immune-related AEs and even fatal events) are not incorporated into Table 3 or model inputs. AE-associated costs (e.g., hospitalization, treatment discontinuation) may significantly impact cost-effectiveness.

Response: Thank you for raising the issue regarding the scope of adverse event (AE) cost inclusion. We agree that serious AEs (grade ≥3), particularly immune-related AEs and fatal events, occurring in clinical trials may generate substantial medical costs and thus have an important impact on cost-effectiveness assessment. In the base-case analysis of this study, we included only grade ≥3 AEs with an incidence rate >5%, primarily based on the following considerations: (1) According to the China Guidelines for Pharmacoeconomic Evaluations and relevant international guidelines, pharmacoeconomic models typically include AEs with a relatively high incidence (e.g., ≥5%) and a significant impact on costs or utilities to ensure model parsimony and operability; (2) In clinical practice, AEs with a low incidence rate (<5%) generally have a limited impact on overall costs and utilities; (3) The data source for the current model is a phase III clinical trial report, and for AEs with low incidence rates, their cost data are often difficult to estimate accurately.

Furthermore, we have addressed this issue in the Limitations section of the Discussion as follows: only severe adverse reactions (≥ Grade 3) with an incidence rate difference of ≥3% between the two treatment groups were included in the analysis. Not all AEs were considered, and the associated management costs may differ from actual clinical practice. However, one-way sensitivity analysis demonstrated that the cost of AE management had minimal impact on the overall results.
3、Patient Flow and Discontinuations：The Materials & Methods section lacks clarity on:
Sample size: Exact patient numbers randomized to each arm Treatment discontinuations: Rates due to AEs/progression, which affect drug utilization and cost.

Response: Thank you for your careful review. We have supplemented and revised the relevant content according to your suggestions, as detailed below:

(1) A total of 346 patients with advanced hepatocellular carcinoma who had not previously received systemic therapy were enrolled in this trial and randomly assigned in a 2:1 ratio to the finotonlimab plus bevacizumab biosimilar group (230 patients) or the sorafenib group (116 patients). This sample size information has been added to section "2.1 Target population and treatment regimen."
(2) Discontinuation due to disease progression: This is directly reflected by the progression-free survival (PFS) curve. Once patients enter the progressed disease state, they no longer continue the original treatment regimen. We have supplemented the subsequent treatment costs based on the different drugs used in the clinical trial (immunotherapy, targeted therapy, chemotherapy) and their respective proportions.
(3) Discontinuation due to adverse events: According to the clinical trial report, 46 patients in the experimental group discontinued treatment due to adverse events, compared with 4 patients in the control group. In the model specification, we assume that patients who discontinue treatment due to adverse events stop receiving the study drug but remain in the progression-free state, and the corresponding costs of managing the adverse events are incurred.
    4、Some figures (particularly Figure 2) currently suffer from poor readability due to:small font sizes for axis labels and legends

Response: Thank you for your careful review. Following your suggestion, we have enlarged the font of the axis labels and legend in Figure 2. The revised figure has been replaced in the manuscript.
