# Supplementary material for: Economic evaluation of finotonlimab plus bevacizumab as first-line therapy for advanced hepatocellular carcinoma
Source: PLoS One. 2026 May 14;21(5):e0349044. doi: 10.1371/journal.pone.0349044 (PMC13175363; doi:10.1371/journal.pone.0349044)
Supplement: Attachment — Submitted filename: Response_to_comments_auresp_2.docx [file pone.0349044.s003.docx]

Reviewer #1: This study still contains obvious errors; please treat academic research with due seriousness. It is recommended that three times the per capita GDP be employed as the willingness-to-pay threshold, i.e. 99,800 × 3 = 299,400. What was the author’s intention in writing ‘three times per capita GDP’ as ‘two times per capita GDP’ (199,600)? (At a WTP threshold of three times China’s 2025 per capita GDP (¥199,600), the probability of the dual-agent regimen being cost-effective was 0%.)

Response: Thank you for pointing out this critical issue. Based on your suggestion, we have revised the threshold to "three times the per capita GDP" throughout the manuscript, with the corresponding willingness-to-pay threshold set to 299,400 CNY. The probability of the two-drug regimen being cost-effective at this threshold has been recalculated. The revised results show that "when using three times the per capita GDP of China (299,400 CNY) as the willingness-to-pay threshold, the probability of the two-drug regimen being cost-effective is 0%."
